# You can go your own way: The targeting signals of trypanosomatid parasites

**DOI:** 10.1371/journal.ppat.1013326

**Published:** 2025-08-01

**Authors:** Max Pendlebury, Julius Lukeš, Michael J. Hammond

**Affiliations:** Institute of Parasitology, Biology Centre, Czech Academy of Sciences, and Faculty of Science, University of South Bohemia, České Budějovice (Budweis), Czech Republic; University of Wisconsin Medical School, UNITED STATES OF AMERICA

## Overview

**Fig 1:**
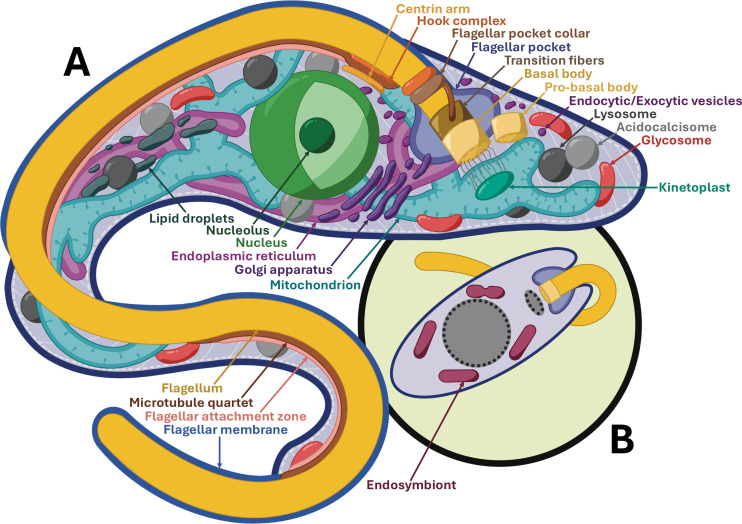
Schematic representation of trypanosomatid model organisms. (A) Overview of *Trypanosoma brucei* procyclic form organelles and cellular compartments. (B) Simplified overview of endosymbiont-containing *Novymonas esmeraldas.*

Targeting and directing cytosol-synthesized proteins into organelles and cellular compartments constitutes a universal eukaryotic challenge. The discovery that specific peptide sequences are responsible for this localization not only earned a Nobel prize, but also provided a powerful tool for investigating eukaryotic evolution and diversification [1]. While the term ‘targeting signal’ encompasses a broad range of sequences with varying properties, here we define them as sequences—or a set of sequences—that are both necessary and sufficient to ensure a protein reaches its correct cellular localization. Parasitic trypanosomatids, represented primarily by model protist *Trypanosoma brucei*, constitute the most thoroughly investigated eukaryotes in this regard outside of opisthokonts and plants. Here we provide a protistan perspective on the targeting signals, both innovated and derived, employed by trypanosomatid flagellates for this purpose (Fig 1).

## The secretory pathway

**Fig 2:**
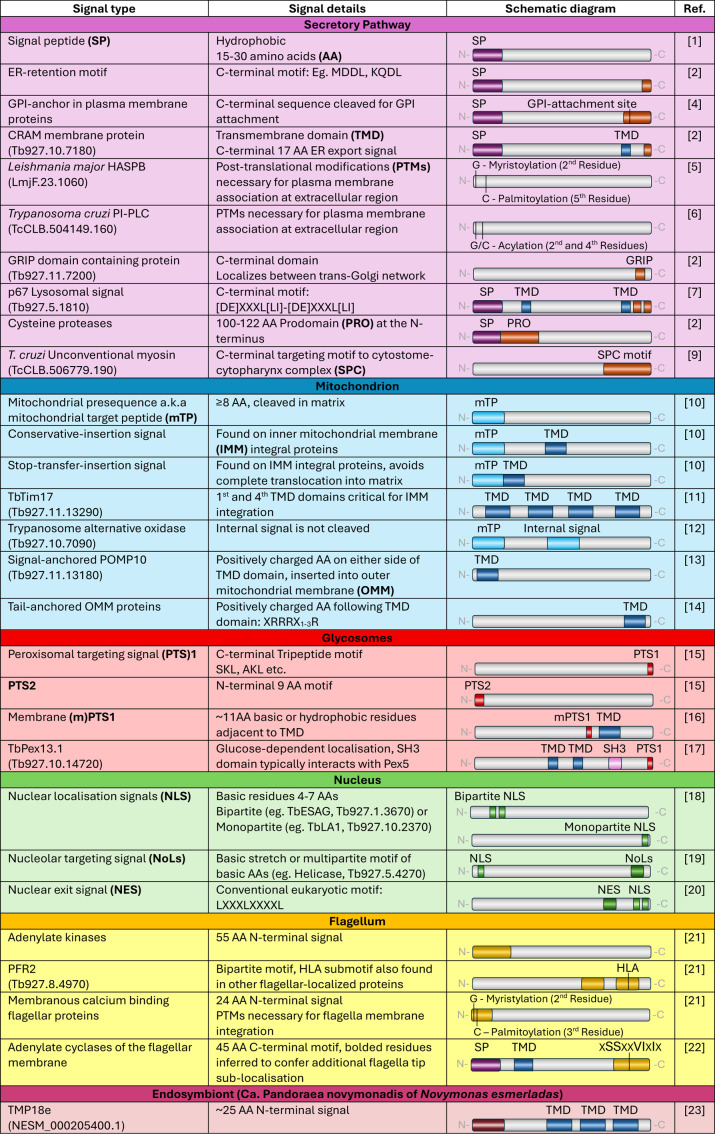
Trypanosomatid signals employed for protein targeting, with accession identity specified in cases of singular examples and acronyms bolded at first appearance. Trypanosomatid species is clarified and specified for protein representatives outside of *Trypanosoma brucei*.

**Fig 3:**
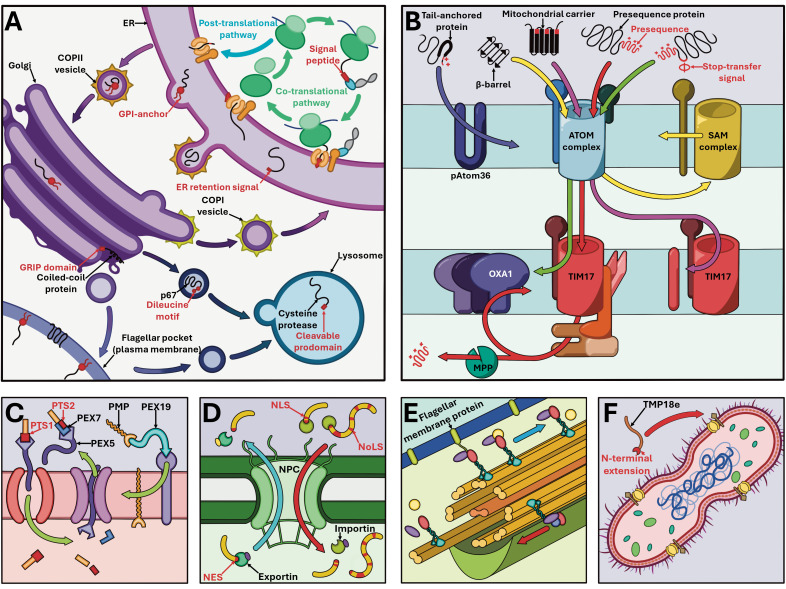
Overview of major protein targeting pathways in *Trypanosoma brucei* (unless stated otherwise). **(A)** Secretory pathway with relevant organelles of endoplasmic reticulum (ER), Golgi body, lysosomes, and plasma membrane. **(B)** Protein targeting across and into both mitochondrial membranes. **(C)** Targeting mediated by PEX proteins in glycosomes, employed on proteins containing PTS1 and PTS2 targeting signals as well as peroxisomal membrane proteins (PMP). **(D)** Trafficking through the nuclear pore complex (NPC), with entry mediated *via* Nuclear localization signals (NLS) and Nucleolar targeting signals (NoLS) and exit *via* Nuclear exit signals (NES). **(E)** Flagellar protein trafficking, with retrograde (blue arrow) and anterograde direction (red arrow) indicated across the axoneme. **(F)** Endosymbiont-targeted TMP18e in *Novymonas esmeraldas*. Targeting signals on proteins are highlighted in red, with acronyms defined in Fig 2.

The eukaryotic signal peptide (SP), a 15–30 amino acid (AA)-long hydrophobic sequence conferred to the N-terminus (Fig 2), designates newly synthesized proteins for the secretory pathway, which encompasses several organelles (Fig 3A) [1]. Proteins are imported into the endoplasmic reticulum (ER) co-translationally through a signal recognition particle-dependent pathway, or post-translationally without this complex. Confinement to the ER is delineated by a tetrapeptide retention motif, mildly divergent from those found in opisthokonts (Fig 2) [2]. *T. brucei* SP-containing proteins are unlike opisthokonts in that they can utilize both pathways of transit, rather than being transported solely through the co-translational pathway. However, glycosylphosphatidylinositol (GPI)-anchored proteins, which typically possess SPs, have been shown strictly utilizing the post-translational pathway for transport [3].

*T. brucei* expresses primarily GPI-anchored proteins at the cell surface, including variable surface glycoproteins (VSGs) which are critical to parasite virulence in the mammalian host. As a result, the secretory pathway is tailored for the bulk flow of GPI-anchored proteins to the cell membrane. In the ER, a C-terminal hydrophobic sequence is cleaved and replaced with GPI (Fig 2), which then acts as a targeting signal to the cell membrane. All folded proteins are exported from the ER in coat protein II (COPII) vesicles, while GPI-anchored VSGs rely on a distinct type of COPII vesicle to transport this cargo exclusively. This likely represents a mechanism for the priority export of these abundant VSGs, which arrive at the surface faster relative to other membrane proteins [4]. Other polytopic membrane proteins are exported (directly or indirectly) *via* alternate targeting signals, such as that of the cysteine-rich, acidic, integral membrane (CRAM) protein, which relies on a hydrophilic C-terminal sequence for both ER export and cell membrane localization (Fig 2) [2]. Other trypanosomatids display unique cell-membrane targeting signals which are dependent on post-translational modifications of key residues. In *Leishmania major*, a hydrophilic acylated surface protein B (HASPB) lacks transmembrane domains (TMDs), a SP, and a GPI anchor, instead relying on two acylation sites at the N-terminus which are sufficient for cell surface localization [5]. Additionally, *Trypanosoma cruzi*-specific phosphoinositide phospholipase C (TcPI-PLC) localizes to the plasma membrane during the amastigote stage, only when residues Gly2 and Cys4 are acylated [6].

Targeting of proteins to the Golgi and lysosomes in trypanosomatids is similar to that of opisthokonts. Selected proteins are targeted to specific regions within the Golgi apparatus, as seen with coiled-coil proteins possessing a Golgin-97, RanBP2alpha, Imh1p, and p230/golgin-245 (GRIP) domain, which are targeted exclusively to the *trans*-cisternae [2]. Secretory proteins can be further sorted for transport to the lysosomes, with lysosomal membrane protein p67 directed from the Golgi apparatus utilizing two dileucine motifs, deletion of which results in cell membrane trafficking instead (Fig 2) [7]. Cysteine proteases of the lysosomal lumen rely on a cleavable N-terminal prodomain in addition to a SP for trafficking (Fig 2) [2]. These targeting signals also appear to be conserved within the stage-specific megasomes and multivesicular tubule lysosomes of *Leishmania* species [8]. For other lysosome-like organelles, including acidocalcisomes and *T. cruzi*-specific reservosomes, little is known concerning protein targeting, though there is evidence to suggest that trafficking to acidocalcisomes differs between trypanosomatids and opisthokonts [2].

*T. cruzi* additionally possesses a derived feeding apparatus known as the cytostome-cytopharynx complex (SPC) which acts as its main site for endocytosis. Though the structure itself is enigmatic, a class of orphan myosins employ a C-terminal extension which is sufficient for SPC targeting (Fig 2) [9].

## Mitochondrion

Despite possessing radically diverged mitochondrial translocation machinery when compared to other eukaryotes [10], mitochondrial targeting signals in *T. brucei* have remained relatively consistent and recognizable (Fig 3B).

Many *T. brucei* matrix and inner mitochondrial membrane (IMM) proteins rely on positively charged N-terminal presequences (Fig 2). Typically shorter than in other eukaryotes (8 ≤ AA), they interact with diverged outer mitochondrial membrane (OMM) atypical translocase of outer membrane (ATOM) complex, then with TbTim17 in the IMM to translocate into the matrix where the presequence is cleaved (Fig 3B) [10]. The IMM proteins possessing a hydrophobic stretch following this presequence will arrest within TbTim17 and laterally release into the IMM, while the IMM proteins fully translocated into the matrix are inserted *via* oxidase assembly protein 1 orthologues (Fig 3B) [10]. The internal signals employed by polytopic transporters, such as mitochondrial carrier proteins, remain sparsely investigated. However, TbTim17 itself employs two internal targeting signals within its first and fourth TMDs for IMM integration (Fig 2) [11]. Trypanosome alternative oxidase additionally complements its presequence with an internal targeting signal, which alone proves sufficient for mitochondrial import (Fig 2) [12].

A sole signal-anchored OMM protein, present in the outer mitochondrial membrane proteome 10 (POMP10), has been characterized. It flanks its N-terminal TMD with canonical positive residues and is reliant on trypanosomatid pATOM36 for insertion in the absence of a mitochondrial import complex (Fig 2) [13]. Similarly, several tail-anchored OMM proteins have recently been experimentally identified. They possess a C-terminal TMD which is succeeded by several basic residues, reminiscent of their opisthokont counterparts (Fig 2) [14]. Contrastingly, the OMM β-barrel proteins of trypanosomatids lack the C-terminal hydrophobic β-hairpin, typically necessary for transport and insertion, suggesting the presence of a divergent targeting mechanism.

## Glycosomes

Trypanosomatids have modified peroxisomes termed glycosomes, which compartmentalize the first six or seven steps of glycolysis. This is achieved by furnishing these enzymes with canonical peroxisomal targeting signal (PTS)1 or PTS2 (Fig 3C). The PTS1 signal is positioned at the C-terminus with a conserved tripeptide motif which interacts with the soluble peroxisome biogenesis factor (PEX)5 chaperone, while PTS2 signal is N-terminally located with a 9 AA-long conserved motif, interacting with both PEX7 and PEX5 (Fig 2) [15].

Most glycosomal membrane proteins contain a membrane PTS1 signal (mPTS1). The *T. brucei* mPTS1 signal includes an ~ 11 AA-long string of hydrophobic or basic residues adjacent to a TMD, as observed in other eukaryotes [16]. However, membranous TbPex13.1 uniquely possesses both a PTS1 as well as a canonical PEX19 binding domain (Fig 2). In glucose-rich environments, mPTS1 mediates insertion into the glycosomes through interaction with PEX19. However, in glucose-poor environments, PTS1 directs TbPex13.1 to the ER, where it is instead involved in de novo glycosome biogenesis [17].

## Nucleus

Trypansosomatids contain a canonical nuclear localization signal (NLS) which can be monopartite, composed of four or seven basic AAs, as well as bipartite with two basic stretches separated by a 10–12 AA-long linker (Fig 2). The nuclear proteome of *T. brucei* is predicted to contain 68% canonical NLSs [18]. The remaining proteins likely rely on a non-canonical NLS or may be imported *via* a complex which contains at least one canonical NLS (Fig 3D). Various *Leishmania* and *T. cruzi* proteins are reported to possess non-canonical NLS of varying properties, highlighting the heterogenous nature of NLSs in trypanosomatids [18].

Nucleolar targeting signals (NoLSs), like NLSs, are dependent on the presence of basic AAs. While NoLS can form a homopolymer string, the overall basicity of the protein is ultimately important for nucleolar localization, leading to multipartite basic motifs as well (Fig 2). As in other eukaryotes, high intrinsic disorder and low hydrophobicity are also important features for nucleolar proteins in *T. brucei* [19]. Trypanosomatids additionally make use of canonical nuclear exit signals similar to those found in opisthokonts to export proteins from the nucleus to the cytoplasm (Fig 2) [20].

## Flagellum

Intraflagellar transport (IFT) complexes are employed by eukaryotes for delivery of many flagellar proteins (Fig 3E). Employed motifs mediating interactions with these IFTs are highly variable, lacking the more unified signals used across membranous organelles.

Two trypanosome adenylate kinases employ a conserved 55 AA-long N-terminal motif which produces a flagellar localization (Fig 2). By contrast, paraflagellar rod 2 (PFR2) protein carries two motifs within its C-terminus: first, a region of approximately ≤56 AA, which by itself incorporates PFR2 within the flagellum as well as the cytoplasm, and a second downstream seven AA-long region which together confers an exclusive flagellar localization (Fig 2) [21]. A specific region of PFR2’s second motif, the tripeptide ‘HLA’, is additionally observed in other flagellar proteins such as TrypARP, as well as in Tektin C, and is required by these proteins to reach the flagellum. By itself, however, this tripeptide is insufficient to produce an exclusive flagellar signal, further demonstrating the multipartite nature of many signals for structural proteins of the flagellum [21].

A number of trypanosomatid membranous flagellar proteins rely on acylation of N-terminal glycine and cysteine with myristate and palmitate, respectively, for proper localization, including small myristoylated protein-1, calflagins and flagellar calcium-binding protein (Fig 2) [21]. A set of adenylate cyclases localizes to varying sub-compartments of the flagellum. Adenylate cyclases possess both a SP and TMD for membrane targeting but also employ a ~ 45 AA-long C-terminal domain for flagellar localization. A selection of five AAs within this region additionally appear responsible for directing certain adenylate cyclases to the flagellar tip (Fig 2) [22].

Specific targeting signals for proteins of the basal body, transition fibers, or the array of cytoskeletal structures that surround the flagellar pocket remains poorly understood by contrast. Given the importance of these structures for parasite virulence, this knowledge gap warrants further investigation.

## Endosymbionts

Two trypanosomatid lineages harbor metabolically beneficial bacterial endosymbionts, into which endosymbiont-targeted proteins (ETPs) encoded by the host are directed (Fig 1B). *Novymonas esmeraldas* targets endosymbiont-associated transmembrane protein 18 (TMP18e) to the membrane of the bacterium Ca*. Pandoraea novymonadis via* a ~ 25 AA-long N-terminal extension, where it controls endosymbiont positioning and copy number, while the ancestral homolog TMP18, which lacks this extension, is targeted instead to the host nuclear envelope (Fig 3F) (Fig 2) [23]. *Angomonas deanei* harbors a singular Ca. *Kinetoplastibacterium* sp. which undergoes coordinated replication prior to that of the host organelles. One bacterial gene, ornithine cyclodeaminase, has undergone lateral gene transfer to the host genome, and is retargeted to the glycosomes *via* a PTS1 signal [24]. At least seven ETPs are observed either at the endosymbiont envelope, division site or cytosol. Available information on targeting motifs for *Angomonas* ETPs is limited, but they are presumed to be delivered *via* Golgi-derived vesicles, despite lacking canonical SPs [24].

## Concluding statements

Many questions and targeting signals remain to be investigated for trypanosomatids, including those for their defining features, such as the kinetoplast or paraflagellar rod. Discoveries pioneered in trypanosomatid biology have often mediated their later observation in opisthokonts and other eukaryotes [25]. In turn, we hope to encourage further research into the intricacies of protein targeting in these paradigmatic parasites.
